# Why does early childhood deprivation increase the risk for depression and anxiety in adulthood? A developmental cascade model

**DOI:** 10.1111/jcpp.13205

**Published:** 2020-02-06

**Authors:** Dennis Golm, Barbara Maughan, Edward D. Barker, Jonathan Hill, Mark Kennedy, Nicky Knights, Jana Kreppner, Robert Kumsta, Wolff Schlotz, Michael Rutter, Edmund J.S. Sonuga‐Barke

**Affiliations:** ^1^ Centre for Innovation in Mental Health School of Psychology University of Southampton Southampton UK; ^2^ Social, Genetic & Developmental Psychiatry Centre Institute of Psychiatry, Psychology and Neuroscience King's College London London UK; ^3^ Department of Psychology Institute of Psychiatry, Psychology & Neuroscience King's College London London UK; ^4^ School of Psychology & Clinical Language Sciences University of Reading Reading UK; ^5^ Department of Child and Adolescent Psychiatry Institute of Psychiatry, Psychology and Neuroscience King's College London London UK; ^6^ Department of Psychology Centre for Clinical and Community Applications of Health Psychology University of Southampton Southampton UK; ^7^ Department of Genetic Psychology Faculty of Psychology Ruhr University Bochum Bochum Germany; ^8^ Max Planck Institute for Empirical Aesthetics Frankfurt Germany; ^9^ Department of Child & Adolescent Psychiatry Aarhus University Aarhus Denmark

**Keywords:** Institutional deprivation, depression, anxiety, emotional problems, developmental cascades, neurodevelopmental problems, longitudinal, prospective, natural experiment

## Abstract

**Background:**

Using data from the English & Romanian Adoptees (ERA) study, we recently reported that early time‐limited exposure to severe institutional deprivation is associated with *early*‐onset and persistent neurodevelopmental problems and *later*‐onset emotional problems. Here, we examine possible reasons for the late emergence of emotional problems in this cohort. Our main focus is on testing a developmental cascade mediated via the functional impact of early‐appearing neurodevelopmental problems on late adolescent functioning. We also explore a second putative pathway via sensitization to stress.

**Methods:**

The ERA study includes 165 Romanian individuals who spent their early lives in grossly depriving institutions and were subsequently adopted into UK families, along with 52 UK adoptees with no history of deprivation. Age six years symptoms of neurodevelopmental problems and age 15 anxiety/depression symptoms were assessed via parental reports. Young adult symptoms of depression and anxiety were assessed by both parent and self‐reports; young adults also completed measures of stress reactivity, exposure to adverse life events, and functioning in work and interpersonal relationships.

**Results:**

The path between early institutional deprivation and adult emotional problems was mediated via the impact of early neurodevelopmental problems on unemployment and poor friendship functioning during the transition to adulthood. The findings with regard to early deprivation, later life stress reactivity, and emotional problems were inconclusive.

**Conclusions:**

Our analysis suggests that the risk for adult depression and anxiety following extreme institutional deprivation is explained through the effects of early neurodevelopmental problems on later social and vocational functioning. Future research should more fully examine the role of stress susceptibility in this model.

## Introduction

Early institutional deprivation is associated with a range of later psychopathology (Bos et al., 2011; Woodhouse, Miah, & Rutter, 2017). Emotional difficulties might be expected to be prominent among these risks: Both psychological theories that assign a foundational role to primary carer relationships (Bakermans‐Kranenburg et al., 2011) and biological models of stress system programming through early life adversity (Koss & Gunnar, 2018) predict increased rates of emotional difficulties following institutional deprivation. Consistent with these views, elevated rates of internalizing problems have been reported in some institutionalized samples in childhood (e.g., Bos et al., 2011), and follow‐ups in childhood and early adolescence point to persisting risk for emotional difficulties even when children are removed from institutions and placed in foster or adoptive homes (Humphreys et al., 2015).

The recent completion of the young adult follow‐up of the English and Romanian Adoptees (ERA) study has made it possible to examine the longer‐term impact of severe early institutional deprivation on risk for emotional problems in young adulthood. ERA has tracked the development of children who experienced up to 43 months of the grossly depriving conditions of Romanian orphanages in the 1980s before being adopted by UK families. This creates a powerful natural experiment that allows assessment of the effects of early and time‐limited adversity on subsequent development, unconfounded by the ongoing adversity and familial risk for disorder that often affect interpretation of studies of maltreatment within biological families. Against initial expectation, and despite profound impairment in other domains, levels of emotional problems were not elevated in childhood even among those with extended deprivation (Sonuga‐Barke et al., 2017). Even more intriguingly, recently reported analyses of longer‐term trajectories suggested that Romanian adoptees who experienced more than 6 months deprivation displayed a striking rise in emotional problems between adolescence and young adulthood (Sonuga‐Barke et al., 2017) – a much greater increase than seen in the other ERA study groups (i.e., nondeprived UK adoptees and Romanian children exposed to <6 months deprivation). The current paper sets out to understand the reasons for this.

Here, we use a latent path analytic model of the longitudinal data from the ERA study to explore putative pathways from early deprivation to late‐onset emotional problems. Our main focus is on testing a developmental cascade (Masten & Cicchetti, 2010) running via the downstream consequences of the neurodevelopmental problems manifest early in childhood in the ERA sample (Kreppner, O'Connor, Rutter, & English and Romanian Adoptees Study Team, 2001). Most notably, children exposed to more than 6‐months deprivation displayed elevated levels of attention‐deficit/hyperactivity disorder (ADHD; Kreppner et al., 2001), autistic‐type problems (i.e., quasi‐autism; Rutter et al., 2007), and disinhibited social engagement (DSE, O'Connor & Rutter, 2000) – difficulties which proved remarkably persistent through to young adulthood (Sonuga‐Barke et al., 2017). Given the known impact of such disorders on social and educational functioning and achievement (Booster, Dupaul, Eiraldi, & Power, 2012; de Schipper et al., 2016), and the relationship between functional impairment and mental health (McKnight & Kashdan, 2009), it seemed highly plausible that deprivation‐related variants of these conditions could also increase the risk of emotional problems as children encounter the limitations they impose. In the current paper, we examine a number of proximal impairment‐related vulnerability factors acting during the transition from adolescence to adulthood. In particular, guided by past literature on risks for emotional problems (Kendler, Gardner, & Prescott, 2002; La Greca & Harrison, 2005; Paul & Moser, 2009), we explore difficulties in relationship functioning and unemployment as indicators of these more proximal risks.

We also explore a second potential pathway between early childhood deprivation and adult emotional problems. This is mediated via a form of latent vulnerability linked to an underlying deprivation‐related sensitization to stress; the effects of which are only manifest later in life. The possibility that early exposure to adversity can create such latent vulnerabilities within a child, not manifest as disorder until later in the life course, is highlighted by the theory of McCrory and colleagues (McCrory, Gerin, & Viding, 2017). In their model, adversity creates alterations in neuro‐cognitive systems, which may hold adaptive advantages in adverse settings, but function to increase vulnerability to stressors later in life (McCrory et al., 2017). This model provides a cognitive developmental account of the way early adversity can sensitize an individual to later stressful experiences. Deprivation might sensitize biological systems that regulate an individual's response to stress such as the HPA axis (Kumsta et al., 2017), or the emotion processing centers of the brain (Tottenham et al., 2010). From this point of view, the late emergence of emotional problems in the ERA sample could be explained by a deprivation‐related sensitization to stress creating a latent vulnerability affecting mental health as adoptees pass from protective and supportive home environments in childhood and adolescence to potentially more challenging adult settings (Osgood, Foster, & Courtney, 2010). Unfortunately, no data on putative vulnerability biomarkers were collected during the early stages of the ERA study; we can, however, test whether early deprivation leads to a heightened susceptibility to stress in adulthood using self‐ratings of stress reactivity.

## Methods

### Participants

One‐hundred sixty‐five Romanian (91 females – up to 43 months in institutions), 52 UK adoptees (18 females – no deprivation history), and their adoptive families entered the study in the mid‐1990s. Assessments were made at ages 6, 11, and 15 years and in young adulthood (mean age 23.9 years, *SD* = 0.79). Data on the outcomes reported here were available for 206 study members (95%) at age 6 years, 188 (87%) at age 15 years, and for a total of 162 (75%, 142 via parent report and 139 via young adult report) in young adulthood. The majority of the adoptive families were of high (professional, managerial) socioeconomic status (SES). Forty‐two per cent of young adults were living with their parents at the time of the young adult assessments, and the remainder lived in a variety of independent settings; 41% were married/cohabiting; and 12% had their own children.

### Measures

#### Young adult emotional problems

Dimensional measures of adult emotional problems were based on counts of generalized anxiety disorder (GAD) and major depressive disorder (MDD) symptoms created by mapping items from the Conners Comprehensive Behavior Rating Scales (Conners, 2008) – a valid and reliable measure focusing on symptoms over the previous 4 weeks – on to DSM‐5 (American Psychiatric Association, 2013) symptom domains of GAD and MDD. Ratings of 2 and above (on a 0–3 scale) were taken to reflect symptom endorsement. Both parents and young adults completed the questionnaires. Parent reports were chosen as the main outcome measures for the path analyses (a) because more parent than self‐report data were available and (b) to reduce the risk of biases from shared method variance when information about outcomes and mediators are both provided by the same reporters. Importantly, parent‐ and self‐reports were correlated (MDD, *r* = .563, *p* < .001; GAD, *r* = .501, *p* < .001) and both had similar levels of internal consistency (MDD, parent: α = .84, self: α = .81; GAD, parent: α = .85, self: α = .87) and external validity in terms of associations with reports of antidepressant use since age 15 (OR^parent report^ = 2.10, 95% CI = 1.60, 2.73; *p* < .001; OR^self report^ = 1.62, 95% CI = 1.32, 1.98; *p* < .001).

### Potential mediators, moderators and covariates

#### Early childhood deprivation‐related neurodevelopmental problems (age 6)

*ADHD symptoms*: a count of parent‐rated inattention/overactivity symptoms (hyperactivity, sustained attention, distractibility) was taken from the inattention/overactivity subscale of the Revised Rutter Scale (Elander & Rutter, 1996), a well‐established and validated measure of socioemotional and behavioral problems. Symptoms were rated as endorsed if a rating of two (certainly applies) was given on the 0–2 rating scale.

*Autism Spectrum Disorder symptoms (ASD)*: The Social Communication Questionnaire (Rutter, Bailey, & Lord, 2003) is a parent‐completed and clinically validated screen for ASD symptoms that maps onto DSM diagnostic criteria. We used a 15‐item version with five items from each scale (social reciprocal interaction; communication; and repetitive and stereotyped behaviors; see Sonuga‐Barke et al., 2017). Items were rated present (1) or absent (0) (range in the current sample = 0–10, α = .88).

*Disinhibited social engagement (DSE):* assessed from interviewers' ratings of parents' answers to questions about interactions with strangers (Sonuga‐Barke et al., 2017), tapping the constructs of being ‘too friendly’, showing ‘inappropriate intrusiveness’, and being ‘unaware of social boundaries’. Ratings indicating definite evidence of disinhibition in each area were summed to give a total DSE score (range 0–3, α = .71).

#### Adolescence

*Adolescent symptoms of anxiety and depression* were assessed via parent reports on the CAPA (Angold & Costello, 1995) at age 15 years, modified to code symptoms over the period from 11 to 15 years of age (English & Romanian Study Team, 2010).

*Adoptive family SES* was assessed via parental occupational status at the age 15 contact, coded according to the Registrar General's classification of occupations (OPCS, 1980).

#### Young adulthood

*Stress reactivity* was assessed by young adult self‐reports using the Perceived Stress Reactivity Scale (Schlotz, Yim, Zoccola, Jansen, & Schulz, 2011), a 23‐item scale measuring different aspects of self‐perceived reactivity to everyday stress. It has good test–retest reliability (Schlotz et al., 2011) and validity (Schlotz, Hammerfald, Ehlert, & Gaab, 2011).

*Negative life events ages 15–24:* Exposure to potentially stressful life events was assessed by young adults' responses to a 25‐item Impact of Life Events Questionnaire (Crane et al., 2016). We summed reported exposure to 14 negative events (deaths of close family members or friends; serious illnesses among close family members, friends or the study participant; breakdowns of close relationships; problems at work or in educational settings) experienced between adolescence and young adulthood.

*Love Relationships and Friendships* in young adulthood were assessed using the Revised Adult Personality Functioning Assessment (RAPFA; Hill et al., 2008), an investigator‐based interview that assesses domain‐specific functioning over the previous five years (here, ages 19–24). The Love Relationships scale examines functioning in intimate, exclusive relationships and includes assessments of making and maintaining relationships, and the presence of features such as discord, confiding, and support. The Friendships scale focuses on similar features in relationships with friends, that is, relationships that are specific but not exclusive. Interviews were audio‐recorded and detailed summaries prepared from the tapes. Ratings in each domain range from 1 (a high level of adaptation) to 9 (very poor functioning) over the rating period. In the ERA study, the RAPFA interviews were conducted by psychology graduates (authors MK and NK) with prior experience of clinical/research interviewing. The interviewers were trained in the administration and rating of the RAPFA by its developer (author JH). Inter‐rater reliability was high, with intraclass correlations of *r* = .96 for Love Relationships and *r* = .88 for Friendships, based on a randomly selected 20% of transcripts.

*Months unemployed ages 19–24:* number of months of unemployment in the five years preceding the young adult follow‐up was recorded as part of the RAPFA (Hill et al., 2008). Periods when individuals were in either part‐time or full‐time education were excluded from this calculation.

### Procedure

Ethical approval for the young adult follow‐up was received from the University of Southampton Research Ethics Committee. At each wave, all participants (adoptees and their parents) gave written informed consent or verbal assent, as developmentally appropriate. The main assessments took place in participants' homes; interviewers received full training in the administration of all interview modules and were blind to participants' background and placement histories. Some questionnaire measures were completed online or returned by post at the young adult follow‐up.

### Statistical analysis

As in previous analyses of ERA data, we divided the Romanian adoptee sample into two groups: those with up to six months institutional deprivation (Rom < 6 m, *n* = 67 at entry to the UK, including 21 adoptees placed directly from their homes) and those who spent between 6 and 43 months in the institutions (Rom > 6 m*,* *n* = 98 at entry). Prior analyses have validated this distinction by showing a step‐change in risk within the Romanian group between those exposed to 6‐ and 12‐month deprivation (Kreppner et al., 2007).

The analyses proceeded in three main steps. First, we compared the deprivation groups on levels of young adult emotional problem symptoms. In Step 2, we explored potential mediators of associations with young adult emotional problems through (a) comparison of proposed mediators across the deprivation groups and (b) tests of associations between proposed mediators and young adult emotional symptoms. These tests used logistic regression, ordinal logistic regression, ordinary least squares regression, or negative binomial regression as appropriate to the distribution of the dependent variable of interest. We cite odds ratios (OR), β coefficients and incident rate ratios (IRR), along with 95% confidence intervals (CI), as appropriate. Finally, in Step 3, we took forward all variables associated with both institutional deprivation and emotional problems and used latent path analytic models to test potential mediators. Full details of the specific models tested are given below. Potential mediators were entered as indirect effects and programmed in model constraint statements in Mplus (Muthén & Muthén, 1998–2017). All indirect effects were bootstrapped 10,000 times with 95% bias‐corrected confidence intervals. To establish model fit, we used the chi‐square statistic (good fit: nonsignificant value); the comparative fit index and Tucker–Lewis index (CFI & TLI; good fit >0.95); and root‐mean‐square error of approximation (RMSEA; good fit <0.05). Model parameters were estimated via maximum likelihood estimation with robust standard errors. Analyses for Steps 1 and 2 were undertaken in Stata version 15 (StataCorp, 2015); the path analysis was conducted in Mplus version 8.3 (Muthén & Muthén, 1998–2017).

Missing data and participant dropout were largely consistent with an assumption of data missing completely at random (Sonuga‐Barke et al., 2017). The one exception was parent reports of young adult emotional problem symptoms, where higher response rates were predicted by lower ASD scores at age 6 and higher family SES and parent‐reported adolescent anxiety symptoms at age 15. We created inverse probability weights (Seaman & White, 2013) based on these factors and repeated the analyses for Steps 1 and 2 using the weights to assess the impact of this differential response (see Results). In Step 3, we conditioned the model on family SES and age 15 anxiety/depression symptoms as well as on sex; we note that inclusion of these variables in the analysis can help to minimize bias and maximize recoverability of ‘true’ scores (Little & Rubin, 2002).

## Results

### Group differences in emotional symptoms and disorders in young adulthood

**Figure 1:**
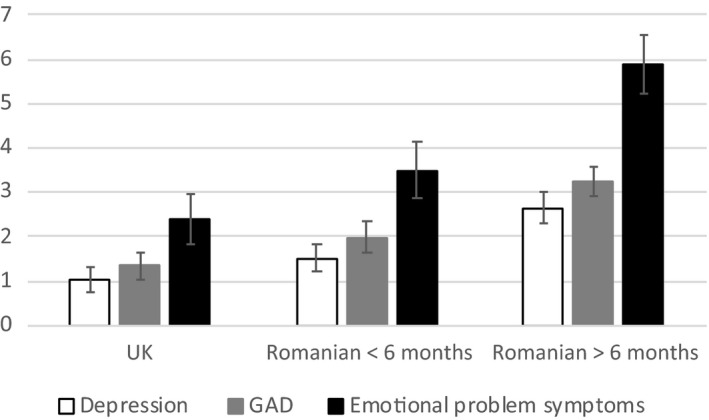
Parent‐rated emotional problem symptoms: age 24 (Conners Conprehensive Behavior Rating Scales)

Figure 1 shows mean levels of parent‐rated young adult depression and GAD symptoms in the three deprivation exposure groups. Depression and GAD symptom ratings were highly correlated (*r* = .86, *p* < .001); Figure 1 also shows mean scores on a combined indicator of emotional problem symptoms in young adulthood. After including biological sex as a covariate, all measures showed a clear pattern of group differences: young adults who had experienced the most extended exposure to institutional deprivation (Rom > 6 months) had higher levels of emotional symptoms than those in both the UK and the Rom < 6 m groups, while these two groups did not differ (Table 1). Weighted group comparisons were closely similar (Table S1), and differences between the Rom > 6 m and UK groups in levels of young adult emotional problems remained when adjusted for levels of adolescent depression and anxiety symptoms in adolescence (Depression: IRR = 1.82, CI = 1.02, 3.28, *p* = .043; GAD: IRR = 1.76, CI = 1.09, 2.84, *p* = .021; general emotional problems: IRR = 1.80, CI = 1.07, 3.02, *p* = .026). Group comparisons on young adult reports of MDD and GAD symptoms followed a similar pattern (Table 1). As the proximal mediators we planned to examine derived from young adult reports, we focused on parent ratings of emotional problem symptoms as the main outcome in the remainder of the analyses to avoid problems of shared variance.

**Table 1:** Early deprivation and young adult emotional symptoms

	Deprivation group			Group contrasts^a^		
	UK	Rom < 6 months	Rom > 6 months	Rom < 6 months vs. UK (IRR, 95% CI)	Rom > 6 months vs. UK (IRR, 95% CI)	Rom > 6 months vs. Rom < 6 months (IRR, 95% CI)
Parent reports	(*n* = 38)	(*n* = 45)	(*n* = 60)			
Symptom counts (CBRS)^b^	**Mean (*SD*)**	**Mean (*SD*)**	**Mean (*SD*)**			
Depression	1.03 (1.84)	1.51 (2.10)	2.65 (2.60)	1.38 (0.71, 2.68)	2.43 (1.40, 4.49)**	1.76 (1.02, 3.05)*
GAD	1.35 (1.88)	1.98 (2.36)	3.24 (2.68)	1.37 (0.81, 2.31)	2.27 (1.40, 3.68)**	1.66 (1.08, 2.56)*
Emotional problem symptoms (Depression + GAD)	2.39 (3.53)	3.49 (4.31)	5.89 (5.07)	1.37 (0.77, 2.42)	2.34 (1.38, 3.96)**	1.71 (1.05, 2.78)*
Young adult reports	(*n* = 33)	(*n* = 39)	(*n* = 52)			
Symptom counts (CBRS)^b^	**Mean (*SD*)**	**Mean (*SD*)**	**Mean (*SD*)**			
Depression	2.10 (2.30)	2.31 (2.47)	3.34 (2.58)	1.09 (0.67, 1.76)	1.57 (1.00, 2.45)*	1.44 (0.95, 2.18)†
GAD	1.94 (2.47)	2.67 (2.76)	3.70 (2.68)	1.36 (0.80, 2.31)	1.87 (1.14, 3.06)*	1.37 (0.87, 2.16)
Emotional problem symptoms (Depression + GAD)	4.10 (4.51)	4.98 (5.04)	7.04 (5.05)	1.21 (0.73, 2.03)	1.72 (1.06, 2.77)*	1.41 (0.90, 2.22)

a Covaried for sex.

b CBRS, Conners Comprehensive Behavior Rating Scales (past 4 weeks).

^†^ *p* < .1.

* *p* < .05.

** *p* < .01.

### Identifying potential mediators

Levels of age 6 neurodevelopmental problems (inattention/overactivity, autism spectrum, and DSE symptoms) were all higher in the Rom > 6 months than the UK and Rom < 6 months groups (Table 2). During the transition to adulthood (ages 19 to 24 years), the Rom > 6 months group had more extended periods of unemployment than the UK and Rom < 6 months groups, and poorer functioning in love relationships and friendships (primarily reflecting ‘avoidant’ functioning, with limited confiding and support). Perceived stress reactivity was also somewhat greater in the Rom > 6 group although the effects did not reach statistical significance. Furthermore, a supplementary path analysis provided no evidence that perceived stress reactivity mediated the pathway from early deprivation to young adult emotional difficulties (see Table S2). There were no group differences in negative life event exposure in adolescence/early adulthood. Parent‐rated young adult emotional problems were associated with all potential mediators (Table 3; for weighted associations see Table S3). Rates of young adult emotional problems were unrelated to family SES at age 15 or to the young adults' living situations at the time of the follow‐up, their marital/cohabiting status, or whether they had children (Table S4).

**Table 2:** Potential mediators: Associations with deprivation

	Deprivation group			Group contrasts		
	UK Mean (*SD*)	Rom < 6 months Mean (*SD*)	Rom > 6 months Mean (*SD*)	Rom < 6 months vs. UK (OR/IRR/β, 95% CI)	Rom > 6 months vs. UK (OR/IRR/β, 95% CI)	Rom > 6 months vs. Rom < 6 months (OR/IRR/β, 95% CI)
Early‐onset neurodevelopmental problems (age 6 years)						
Inattention/ overactivity	0.50 (0.48)	0.49 (0.48)	0.88 (0.58)	0.93 (0.49, 1.74)	3.29 (1.79, 6.03)***	3.55 (2.00, 6.31)***
ASD	1.81 (1.97)	1.85 (1.72)	3.02 (2.46)	1.02 (0.71, 1.48)	1.67 (1.20, 2.32)**	1.63 (1.20, 2.21)**
DSE	0.15 (0.54)	0.39 (0.80)	0.83 (1.01)	2.85 (0.96, 8.42)†	8.43 (3.11, 22.87)***	2.96 (1.49, 5.88)**
Adolescent/early adult functioning and exposures						
Perceived stress reactivity	19.68 (8.31)	19.58 (9.99)	23.38 (9.42)	−0.10 (−4.45, 4.26)	3.70 (−0.44, 7.83)	3.80 (−0.21, 7.80)†
RAPFA: Love relationships	3.79 (2.44)	4.29 (2.42)	5.51 (2.71)	1.53 (0.69, 3.38)	3.36 (1.57, 7.16)**	2.19 (1.05, 4.46)*
RAPFA: Friends	2.39 (0.86)	3.28 (1.68)	4.41 (2.41)	3.40 (1.36, 8.51)**	8.01 (3.28, 19.58)***	2.36 (1.14, 4.89)*
Months unemployed	4.03 (6.99)	5.79 (11.72)	12.18 (15.80)	1.44 (0.56, 3.67)	3.03 (1.27, 7.21)*	2.10 (0.93, 4.76)
Negative life events	3.41 (2.11)	3.53 (1.62)	4.00 (2.35)	1.36 (0.62, 2.98)	1.73 (0.82, 3.64)	1.27 (0.64, 2.51)

ASD, Autism spectrum disorder symptoms (Social Communication Questionnaire); DSE, Disinhibited social engagement; RAPFA, Revised Adult Personality Functioning Assessment (ages 19–24 years); Negative life events ages 15–24 years.

^†^ *p* < .10.

* *p* < .05.

** *p* < .01.

*** *p* < .001.

**Table 3:** Potential mediators: Associations with young adult emotional problem symptoms

Outcome	CBRS Emotional problem symptoms (IRR, 95% CI)
Early‐onset neurodevelopmental problems (age 6 years)	
Inattention/overactivity	2.19 (1.49, 3.22)***
ASD	1.26 (1.12, 1.42)**
DSE	1.26 (1.00, 1.60)†
Early adult functioning (age 19–24 years)	
RAPFA love relationships	1.23 (1.13, 1.35)***
RAPFA friends	1.21 (1.07, 1.36)**
Months unemployed	1.04 (1.03, 1.06)***

CBRS, Conners Comprehensive Behavior Rating Scales (parent report) – past 4 weeks. Emotional problem symptoms: depression + GAD; ASD, Autism spectrum disorder symptoms (Social Communication Questionnaire); DSE, Disinhibited social engagement; RAPFA, Revised Adult Personality Functioning Assessment.

^†^ *p* < .1.

** *p* < .01.

*** *p* < .001.

### Testing mediating pathways

As the Rom < 6 months and UK groups did not differ in early adult emotional symptom levels, we combined these groups in the path analysis and modeled deprivation exposure as a binary variable (UK + Rom < 6 months vs. Rom> 6 months). Early neurodevelopmental problems were modeled as a latent factor, comprising the shared variance of the age 6 scores for ADHD, ASD, and DSE symptoms. Young adult GAD and depression symptoms were modeled as a shared variance latent emotional symptoms factor; early adult unemployment and relationship functioning were modeled as proximal mediators; and the outcome and mediators were conditioned on sex, family SES, and age 15 emotional symptoms.

Table 4 shows correlations among the variables. At the bivariate level, early deprivation was associated with the childhood neurodevelopmental problems factor, the young adult emotional problems factor, and all of the proposed mediators; all other variables in the model were also significantly associated. Among the covariates, sex and age 15 anxiety/depression were associated with young adult emotional problems, but family SES was not. In addition, age 15 emotional problems were associated with all of the proposed late adolescent/young adult mediators.

**Table 4:** Bivariate correlations among variables in the path model

Outcome	1.	2.	3.	4.	5.	6.
1. Deprivation	–					
2. Age 6 neurodevelopmental problems^a^	.565***	–				
3. Age 19–24 love relationships	.264***	.314***	–			
4. Ages 19–24 friends	.361***	.484***	.465***	–		
5. Age 19–24 months unemployed	.300***	.326***	.389***	.161*	–	
6. Age 24 emotional problem symptoms^b^	.319***	.519***	.434***	.458***	.560***	–
Covariates						
Sex	‐.069	‐.039	.112	.088	‐.131	‐.200*
Age 15 family SES	.000	.000	.022	‐.025	.071	.007
Age 15 Anxiety/depression^c^	.273***	.154†	.237***	.173*	.435***	.488***

a Latent factor: Inattention/overactivity; Autism spectrum disorder symptoms; Disinhibited social engagement.

b Latent factor: parent‐rated CBRS GAD and depression symptoms.

c Sum of parent‐rated anxiety and depression symptoms (CAPA).

^†^ *p* < .1.

* *p* < .05.

*** *p* < .001.

**Figure 2:**
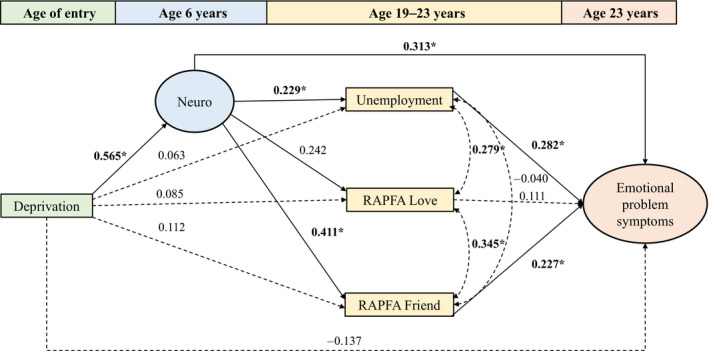
Early adult emotional problem symptoms: mediators of associations with institutional deprivation (conditioned on sex, family SES, and age 15 emotional symptoms) [Colour figure can be viewed at wileyonlinelibrary.com]

The model (Figure 2) showed adequate fit to the data (χ^2^ [31] = 41.62, *p* = .10; CFI = 0.977; TLI = 0.956; RMSEA = 0.050, 90% CI = 0.000, 0.087, *p* = .463). It identified strong associations between deprivation and early neurodevelopmental difficulties, along with significant pathways linking early neurodevelopmental problems with young adult functioning in friendships and the extent of early adult unemployment. Age 15 emotional problems were associated with poorer love relationships and more extended unemployment in early adulthood. Of the proximal mediators, friendship functioning and unemployment showed significant associations with early adult emotional symptoms. With these factors included in the model, the direct pathway from deprivation to early adult emotional problems was no longer significant, though a significant (*p* = .046) link between early neurodevelopmental difficulties and early adult emotional symptoms remained.

Results from tests of the indirect paths are shown in Table 5. There was no direct link between early deprivation and adult emotional symptoms; instead, deprivation predicted neurodevelopmental problems at age 6, and these in turn predicted emotional symptoms directly, and also indirectly via both problems in friendship functioning and the extent of unemployment in late adolescence/early adulthood.

**Table 5:** Indirect effects on early adult emotional symptoms via childhood neurodevelopmental problems and young adult functioning (covaried for sex and age 15 emotional symptoms)

Model	Pre‐adoption	Postadoption		Nonstandardized estimate	Bootstrapping 90% CI		Bootstrapping 95% CI	
		Age 6	Ages 19–24		LL	UL	LL	UL
1	Deprivation	ND problems		0.681	0.249	1.404	0.166	1.635
2	Deprivation	ND problems	Unemployment	0.142	0.030	0.411	0.011	0.489
3	Deprivation	ND problems	Friends	0.204	0.055	0.592	0.022	0.682

ND, neurodevelopmental.

## Discussion

Adoptees in the ERA sample exposed to extended early global deprivation showed no excess risk of emotional problems in childhood and early adolescence, despite marked impairments in other domains (Sonuga‐Barke et al., 2017). Emotional problems began to emerge in this group during mid‐adolescence (Sonuga‐Barke, Schlotz, & Kreppner, 2010), and we recently reported a further increase as the young people transitioned from adolescence to young adulthood (Sonuga‐Barke et al., 2017). The main goal of this follow‐up paper was to explore developmental pathways contributing to these effects.

Associations between institutional deprivation and young adult emotional problems were broad‐based, with adult adoptees exposed to extended deprivation in childhood being at elevated risk for both depression and GAD‐related symptomatology in young adulthood, whereas those who had spent <6 months in the institutions were not. These group differences persisted when covaried for adolescent depression and anxiety symptoms, confirming the exacerbation in risk for emotional difficulties faced by young people with extended exposure to early deprivation during the transition to adulthood.

We explored a range of potential mediators of these effects, guided by the broader literature on risk factors for emotional disorders in adulthood. Our primary focus was on testing the hypothesis that the early‐onset neurodevelopmental problems that were such striking and persistent sequelae of extended institutional rearing in the ERA sample might be implicated in a developmental cascade, with effects on adult emotional problems mediated via their downstream consequences on late adolescent/early adult social functioning. Consistent with this view, we found that a latent neurodevelopmental factor – including age 6 indicators of ADHD and autism spectrum disorder symptoms, and disinhibited social engagement – was strongly associated with more extended institutional deprivation, and in its turn predicted more proximal risks for early adult emotional problems: difficulties in late adolescent/early adult relationship functioning, and also problems in the world of work.

Early‐onset neurodevelopmental disorders are frequently associated with problems in relationship functioning (Hedley, Uljarević, Wilmot, Richdale, & Dissanayake, 2018; Humphreys et al., 2013), so it is no surprise that deprivation‐related variants of these neurodevelopmental problems are also associated with impairment in these domains. In the ERA sample, the emergence of emotional problems at the transition to adulthood, mediated by interpersonal functioning, may suggest that the social supports available to the young people at this stage in the life course were increasingly reliant on their own interpersonal competences rather than those of their adoptive families. Developmental processes in the role of different types of social relationship may explain why friendships rather than romantic functioning were associated with emotional difficulties. Poor friendship quality in late adolescence/early adulthood may create vulnerability as it does during adolescence (La Greca & Harrison, 2005), whereas romantic relationships may not yet have lasted long enough to perform the attachment functions that might protect against emotional difficulties (Hazan & Zeifman, 1999).

Unemployment emerged as a second source of vulnerability for young people with more extended exposure to deprivation. Unemployment has long been identified as a strong correlate of, and likely causal risk factor for, psychopathology (Paul & Moser, 2009), whether via impacts on material resources and financial anxiety, social isolation and loss of self‐esteem, health‐related behaviors, or effects on subsequent employment prospects. Young adults may be especially vulnerable to processes of this kind, and contextual effects may also play a part: in the ERA sample, many of the young people grew up in socioeconomically advantaged adoptive families where – alongside the extensive supports available to them – the social–psychological impact of unemployment may have been particularly accentuated.

With these factors included in the model, a direct path from childhood neurodevelopmental problems to early adult emotional difficulties still remained, suggesting that other mediating factors may also be involved. Testing these was beyond the scope of the current study. We did, however, explore the potential role of stress sensitization in linking early deprivation to late‐onset emotional problems – although our ability to do this was heavily limited by the measures available. This exploration was inspired by the notion, reformulated recently by McCrory and colleagues (McCrory et al., 2017) – that early deprivation might impact neuro‐cognitive systems creating a latent vulnerability to later stress, and so to the eventual manifestation of emotional problems. To start to explore this pathway, we employed self‐rating data from a questionnaire designed to measure individuals' perceived reactivity to stress completed in young adulthood. Although there was a hint of a relationship between deprivation and stress reactivity (more deprivation, greater stress reactivity), there was no evidence that stress reactivity assessed in this way mediated pathways from early deprivation to adult emotional problems. However, this analysis was heavily constrained by the lack of (a) measures of cognitive markers of early vulnerability and (b) a broader range of measures of stress‐related behaviors at multiple time points. As a result, the possibility that the late emergence of emotional problems was due to deprivation‐related latent vulnerability to stress cannot be ruled out definitively. Indeed, we have recently reported long‐term effects of deprivation on HPA axis function (Kumsta et al., 2017), and we have previously found that a variant of the serotonin transporter gene moderated the effect of deprivation on adolescent emotional problems (Kumsta et al., 2010). Integrating neurobiological and genetic studies into pathway models of cascading mental health effects of early deprivation is an important goal of future research.

### Limitations

Our findings should be seen in the light of some limitations. First, though we were successful in collecting some data from 75% of the sample in young adulthood, the modest size of the ERA samples inevitably limited statistical power and may have reduced our capacity to detect some effects. Second, although in general representative of the original sample, response was selective with regard to prior emotional problems, with parents who had reported higher levels of emotional difficulties in their children in adolescence over‐represented in the analyzed sample, and those from lower SES backgrounds, and whose children had displayed higher rates of ASD symptoms at age 6, being under‐represented. We used weights to take some account of these effects in the preliminary analyses and conditioned the path model on SES and parent‐reported emotional difficulties at age 15. Third, because all of the participants in our study were adopted, we were unable to explore the buffering effects of adoption reported in other studies of postinstitutional samples. Fourth, we tested specific, hypothesized mediators of the links between institutional deprivation and young adult emotional difficulties, but other mediating processes may also be implicated. Finally, as mentioned above, our analysis of the potential role of stress sensitization was constrained by the measures available.

### Clinical implications

Our results highlight the risk of the late onset of depression and anxiety in the transition to adulthood among young people who have experienced extended early severe deprivation. In addition to underlining the need for continuing professional care and support across this age period, our findings suggest that specific extra resources should be focused on helping these vulnerable young people to more effectively negotiate the vital life transition between adolescence and adulthood, with a particular focus on establishing and maintaining friendship networks and occupational therapies that promote meaningful engagement with the world of work. In light of the long‐term effects of interpersonal difficulties, social skills training might be a sensible focus for treatment starting earlier in life. Parents should be provided with the skills required to support their adoptive children during these difficult periods into young adulthood. Mental health professionals and primary care workers should be vigilant for mental health problems in young adults who have experienced early deprivation.

## Supporting information


**Table S1.** Associations between early deprivation and parent‐reported young adult emotional problem symptoms (weighted).
**Table S2.** Indirect effects on early adult emotional symptoms via perceived stress sensitivity (covaried for sex and age 15 emotional symptoms).
**Table S3.** Potential mediators: Associations with young adult emotional problem symptoms (weighted).
**Table S4.** Family and individual demographic factors: associations with young adult emotional problem symptoms.

## References

[jcpp13205-bib-0001] American Psychiatric Association (2013). Diagnostic and statistical manual of mental disorders (5th edn). Arlington, VA: American Psychiatric Association.

[jcpp13205-bib-0002] Angold, A. , & Costello, E.J. (1995). A test–retest reliability study of child‐reported psychiatric symptoms and diagnoses using the Child and Adolescent Psychiatric Assessment (CAPA‐C). Psychological Medicine, 25(4), 755–762. 7480452 10.1017/s0033291700034991

[jcpp13205-bib-0003] Bakermans‐Kranenburg, M.J. , Steele, H. , Zeanah, C.H. , Muhamedrahimov, R.J. , Vorria, P. , Dobrova‐Krol, N.A. , … & Gunnar, M.R. (2011). Attachment and emotional development in institutional care: Characteristics and catch‐up. Monographs of the Society for Research in Child Development, 76(4), 62–91. 25242826 10.1111/j.1540-5834.2011.00628.x PMC4166527

[jcpp13205-bib-0004] Booster, G.D. , Dupaul, G.J. , Eiraldi, R. , & Power, T.J. (2012). Functional impairments in children with ADHD: Unique effects of age and comorbid status. Journal of Attention Disorders, 16(3), 179–189. 20876886 10.1177/1087054710383239

[jcpp13205-bib-0005] Bos, K. , Zeanah, C.H. , Fox, N.A. , Drury, S.S. , McLaughlin, K.A. , & Nelson, C.A. (2011). Psychiatric outcomes in young children with a history of institutionalization. Harvard Review of Psychiatry, 19(1), 15–24. 21250893 10.3109/10673229.2011.549773 PMC3445019

[jcpp13205-bib-0006] Conners, K.C. (2008). Conners comprehensive behavior rating scales. Toronto, ON: MHS.

[jcpp13205-bib-0007] Crane, C. , Heron, J. , Gunnell, D. , Lewis, G. , Evans, J. , & Williams, J.M.G. (2016). Adolescent over‐general memory, life events and mental health outcomes: Findings from a UK cohort study. Memory, 24(3), 348–363. 25716137 10.1080/09658211.2015.1008014 PMC4743605

[jcpp13205-bib-0008] de Schipper, E. , Mahdi, S. , de Vries, P. , Granlund, M. , Holtmann, M. , Karande, S. , … & Bölte, S. (2016). Functioning and disability in autism spectrum disorder: A worldwide survey of experts. Autism Research: Official Journal of the International Society for Autism Research, 9(9), 959–969. 26749373 10.1002/aur.1592 PMC5064728

[jcpp13205-bib-0009] Elander, J. , & Rutter, M. (1996). Use and development of the Rutter parents' and teachers' scales. International Journal of Methods in Psychiatric Research, 6(2), 63–78.

[jcpp13205-bib-0010] English and Romanian Study Team (2010). II. Methods and measures used for follow‐up at 15 years of the English and Romanian Adoptee (ERA) study. Monographs of the Society for Research in Child Development, 75(1), 21–47. 20500632 10.1111/j.1540-5834.2010.00549.x

[jcpp13205-bib-0011] Hazan, C. , & Zeifman, D. (1999). Pair bonds as attachments: Evaluating the evidence. In J. Cassidy & P.R. Shaver (Eds.), Handbook of attachment: Theory, research, and clinical applications (pp. 336–354). New York: The Guilford Press.

[jcpp13205-bib-0012] Hedley, D. , Uljarević, M. , Wilmot, M. , Richdale, A. , & Dissanayake, C. (2018). Understanding depression and thoughts of self‐harm in autism: A potential mechanism involving loneliness. Research in Autism Spectrum Disorders, 46, 1–7.

[jcpp13205-bib-0013] Hill, J. , Pilkonis, P. , Morse, J. , Feske, U. , Reynolds, S. , Hope, H. , … & Broyden, N. (2008). Social domain dysfunction and disorganization in borderline personality disorder. Psychological Medicine, 38(01). 10.1017/S0033291707001626 PMC2828321 17892627

[jcpp13205-bib-0014] Humphreys, K.L. , Gleason, M.M. , Drury, S.S. , Miron, D. , Nelson, C.A. , Fox, N.A. , & Zeanah, C.H. (2015). Effects of institutional rearing and foster care on psychopathology at age 12 years in Romania: Follow‐up of an open, randomised controlled trial. Lancet Psychiatry, 2(7), 625–634. 26303560 10.1016/S2215-0366(15)00095-4 PMC4550037

[jcpp13205-bib-0015] Humphreys, K.L. , Katz, S.J. , Lee, S.S. , Hammen, C. , Brennan, P.A. , & Najman, J.M. (2013). The association of ADHD and depression: Mediation by peer problems and parent‐child difficulties in two complementary samples. Journal of Abnormal Psychology, 122(3), 854–867. 24016021 10.1037/a0033895 PMC3806877

[jcpp13205-bib-0016] Kendler, K.S. , Gardner, C.O. , & Prescott, C.A. (2002). Toward a comprehensive developmental model for major depression in women. The American Journal of Psychiatry, 159(7), 1133–1145. 12091191 10.1176/appi.ajp.159.7.1133

[jcpp13205-bib-0017] Koss, K.J. , & Gunnar, M.R. (2018). Annual Research Review: Early adversity, the hypothalamic–pituitary–adrenocortical axis, and child psychopathology. Journal of Child Psychology and Psychiatry, 59(4), 327–346. 28714126 10.1111/jcpp.12784 PMC5771995

[jcpp13205-bib-0018] Kreppner, J.M. , O'Connor, T.G. , & Rutter, M. , & English and Romanian Adoptees Study Team (2001). Can inattention/overactivity be an institutional deprivation syndrome? Journal of Abnormal Child Psychology, 29(6), 513–528. 11761285 10.1023/a:1012229209190

[jcpp13205-bib-0019] Kreppner, J.M. , Rutter, M. , Beckett, C. , Castle, J. , Colvert, E. , Groothues, C. , … & Sonuga‐Barke, E.J.S. (2007). Normality and impairment following profound early institutional deprivation: A longitudinal follow‐up into early adolescence. Developmental Psychology, 43(4), 931–946. 17605526 10.1037/0012-1649.43.4.93

[jcpp13205-bib-0020] Kumsta, R. , Schlotz, W. , Golm, D. , Moser, D. , Kennedy, M. , Knights, N. , … & Sonuga‐Barke, E. (2017). HPA axis dysregulation in adult adoptees twenty years after severe institutional deprivation in childhood. Psychoneuroendocrinology, 86, 196–202. 28982048 10.1016/j.psyneuen.2017.09.021

[jcpp13205-bib-0021] Kumsta, R. , Stevens, S. , Brookes, K. , Schlotz, W. , Castle, J. , Beckett, C. , … & Sonuga‐Barke, E. (2010). 5HTT genotype moderates the influence of early institutional deprivation on emotional problems in adolescence: Evidence from the English and Romanian Adoptee (ERA) study. Journal of Child Psychology and Psychiatry, 51(7), 755–762. 20345836 10.1111/j.1469-7610.2010.02249.x

[jcpp13205-bib-0022] La Greca, A.M. , & Harrison, H.M. (2005). Adolescent peer relations, friendships, and romantic relationships: Do they predict social anxiety and depression? Journal of Clinical Child and Adolescent Psychology, 34(1), 49–61. 15677280 10.1207/s15374424jccp3401_5

[jcpp13205-bib-0023] Little, J.A. , & Rubin, D.B. (2002). Statistical analysis with missing data (2nd edn). Hoboken, NJ: John Wiley & Sons.

[jcpp13205-bib-0024] Masten, A.S. , & Cicchetti, D. (2010). Developmental cascades. Development and Psychopathology, 22(3), 491–495. 20576173 10.1017/S0954579410000222

[jcpp13205-bib-0025] McCrory, E.J. , Gerin, M.I. , & Viding, E. (2017). Annual Research Review: Childhood maltreatment, latent vulnerability and the shift to preventative psychiatry ‐ the contribution of functional brain imaging. Journal of Child Psychology and Psychiatry, 58(4), 338–357. 28295339 10.1111/jcpp.12713 PMC6849838

[jcpp13205-bib-0026] McKnight, P.E. , & Kashdan, T.B. (2009). The importance of functional impairment to mental health outcomes: A case for reassessing our goals in depression treatment research. Clinical Psychology Review, 29(3), 243–259. 19269076 10.1016/j.cpr.2009.01.005 PMC2814224

[jcpp13205-bib-0027] Muthén, L.K. , & Muthén, B.O. (1998–2017). Mplus user's guide (7th edn). Los Angeles: Author.

[jcpp13205-bib-0028] O'Connor, T.G. , & Rutter, M. (2000). Attachment disorder behavior following early severe deprivation: Extension and longitudinal follow‐up. Journal of the American Academy of Child and Adolescent Psychiatry, 39(6), 703–712. 10846304 10.1097/00004583-200006000-00008

[jcpp13205-bib-0029] OPCS (1980). Classification of occupations. London: HMSO.

[jcpp13205-bib-0030] Osgood, D.W. , Foster, E.M. , & Courtney, M.E. (2010). Vulnerable populations and the transition to adulthood. The Future of Children, 20(1), 209–229. 20364628 10.1353/foc.0.0047

[jcpp13205-bib-0031] Paul, K.I. , & Moser, K. (2009). Unemployment impairs mental health: Meta‐analyses. Journal of Vocational Behavior, 74(3), 264–282.

[jcpp13205-bib-0032] Rutter, M. , Bailey, A. , & Lord, C. (2003). SCQ. The social communication questionnaire. Torrance, CA: Western Psychological Services.

[jcpp13205-bib-0033] Rutter, M. , Kreppner, J. , Croft, C. , Murin, M. , Colvert, E. , Beckett, C. , … & Sonuga‐Barke, E. (2007). Early adolescent outcomes of institutionally deprived and non‐deprived adoptees. III. Quasi‐autism. Journal of Child Psychology and Psychiatry, 48(12), 1200–1207. 18093025 10.1111/j.1469-7610.2007.01792.x

[jcpp13205-bib-0034] Schlotz, W. , Hammerfald, K. , Ehlert, U. , & Gaab, J. (2011). Individual differences in the cortisol response to stress in young healthy men: Testing the roles of perceived stress reactivity and threat appraisal using multiphase latent growth curve modeling. Biological Psychology, 87(2), 257–264. 21419825 10.1016/j.biopsycho.2011.03.005

[jcpp13205-bib-0035] Schlotz, W. , Yim, I.S. , Zoccola, P.M. , Jansen, L. , & Schulz, P. (2011). The perceived stress reactivity scale: Measurement invariance, stability, and validity in three countries. Psychological Assessment, 23(1), 80–94. 21280954 10.1037/a0021148

[jcpp13205-bib-0036] Seaman, S.R. , & White, I.R. (2013). Review of inverse probability weighting for dealing with missing data. Statistical Methods in Medical Research, 22(3), 278–295. 21220355 10.1177/0962280210395740

[jcpp13205-bib-0037] Sonuga‐Barke, E.J.S. , Kennedy, M. , Kumsta, R. , Knights, N. , Golm, D. , Rutter, M. , … & Kreppner, J. (2017). Child‐to‐adult neurodevelopmental and mental health trajectories after early life deprivation: The young adult follow‐up of the longitudinal English and Romanian Adoptees study. The Lancet, 389(10078), 1539–1548. 10.1016/S0140-6736(17)30045-4 28237264

[jcpp13205-bib-0038] Sonuga‐Barke, E.J. , Schlotz, W. , & Kreppner, J. (2010). Differentiating developmental trajectories for conduct, emotion, and peer problems following early deprivation. Monographs of the Society for Research in Child Development, 75(1), 102–124. 20500635 10.1111/j.1540-5834.2010.00552.x

[jcpp13205-bib-0039] StataCorp. (2015). Stata statistical software: Release 14. College Station, TX: Author.

[jcpp13205-bib-0040] Tottenham, N. , Hare, T.A. , Quinn, B.T. , McCarry, T.W. , Nurse, M. , Gilhooly, T. , … & Casey, B.J. (2010). Prolonged institutional rearing is associated with atypically large amygdala volume and difficulties in emotion regulation. Developmental Science, 13(1), 46–61. 20121862 10.1111/j.1467-7687.2009.00852.x PMC2817950

[jcpp13205-bib-0041] Woodhouse, S. , Miah, A. , & Rutter, M. (2017). A new look at the supposed risks of early institutional rearing. Psychological Medicine, 48, 1–10. 28637524 10.1017/S0033291717001507

