# Supplementary material for: Why does early childhood deprivation increase the risk for depression and anxiety in adulthood? A developmental cascade model
Source: J Child Psychol Psychiatry. 2020 Feb 6;61(9):1043–53. doi: 10.1111/jcpp.13205 (PMC8597399; doi:10.1111/jcpp.13205)
Supplement: Supplementary file 1 — Table S1. Associations between early deprivation and parent‐reported young adult emotional problem symptoms (weighted). Table S2. Indirect effects on early adult emotional symptoms via perceived stress sensitivity (covaried for sex and age 15 emotional symptoms). Table S3. Potential mediators: Associations with young adult emotional problem symptoms (weighted). Table S4. Family and individual demographic factors: associations with young adult emotional problem symptoms. [file JCPP-61-1043-s001.docx]

**Supporting information – Why does early childhood deprivation increase the risk for depression and anxiety in adulthood? A developmental cascade model – by Golm *et al*.**

**Table S1.** Associations between early deprivation and parent-reported young adult emotional problem symptoms (weighted).

|  | Deprivation group | | | Group contrasts^a^ | | |
| --- | --- | --- | --- | --- | --- | --- |
|  | UK  (n=38) | Rom  <6m  (n=43) | Rom  >6m  (n=58) | Rom<6m vs UK  (IRR, 95% CI) | Rom>6m vs UK  (IRR, 95% CI) | Rom>6m vs Rom<6m  (IRR, 95% CI) |
| Symptom counts (CBRS) |  |  |  |  |  |  |
| Depression | 1.05 (1.83) | 1.52 (2.06) | 2.28 (2.49) | 1.36 (0.69, 2.67) | 2.14 (1.15, 3.96)* | 1.57 (0.97, 2.57)† |
| GAD | 1.38 (2.01) | 2.00 (2.32) | 2.86 (2.53) | 1.29 (0.73, 2.30) | 1.93 (1.16, 3.22)* | 1.50 (1.00, 2.25)† |
| Emotional problem   symptoms  (Depression+GAD) | 2.54 (3.67) | 3.53 (4.22) | 5.27 (4.84) | 1.32 (0.73, 2.39) | 2.02 (1.19, 3.44)** | 1.54 (1.00, 2.36)† |

CBRS: Conners Comprehensive Behaviour Rating Scales.

† p<0.1 *p<0.05 **p<0.01

**^a^** covaried for sex

**Table S2.** Indirect effects on early adult emotional symptoms via perceived stress sensitivity (covaried for sex and age 15 emotional symptoms).

| Model | Pre-adoption | Post-adoption | | Non-standardized  estimate | Bootstrapping 90% CI | | Bootstrapping 95% CI | |
| --- | --- | --- | --- | --- | --- | --- | --- | --- |
|  |  | Age 6 | Age 23 |  | LL | UL | LL | UL |
| 1 | Deprivation |  | Stress sensitivity | 0.055 | -0.001 | 0.138 | -0.012 | 0.156 |

ND: neurodevelopmental

**Table S3.** Potential mediators: Associations with young adult emotional problem symptoms (weighted).

| Outcome | CBRS emotional problem symptoms  (IRR, 95% CI) |
| --- | --- |
| Early onset problems (age 6 years) |  |
| Inattention/overactivity | 2.11 (1.48, 3.03)*** |
| ASD | 1.25 (1.12, 1.40)*** |
| DSE | 1.24 (1.03, 1.48)* |
| Early adult functioning (age 19-23 years) | |
| RAPFA Love relationships | 1.22 (1.13, 1.33)*** |
| RAPFA Friends | 1.21 (1.11, 1.32)*** |
| Months unemployed | 1.04 (1.03, 1.05)*** |

CBRS: Conners Comprehensive Behaviour Rating Scales (parent report) – past 4 weeks. Emotional problem symptoms: MDD + GAD

ASD: Autism spectrum disorder symptoms (Social Communication Questionnaire); DSE: Disinhibited social engagement; RAPFA: Revised Adult Personality Functioning Assessment

† p<0.1 *p<0·05 **p<0·01 ***p<0·001

**Table S4.** Family and individual demographic factors: associations with young adult emotional problem symptoms.

| Demographic factors | CBRS Emotional problem symptoms  (IRR, 95% CI) |
| --- | --- |
| Family demographic factors |  |
| Adoptive family SES (age 15) | 1.09 (0.85, 1.42) |
| Individual demographic factors |  |
| Living with parents, age 24 | 1.14 (0.70, 1.86) |
| Married/cohabiting, age 24 | 0.78 (0.48, 1.29) |
| Has own children, age 24 | 1.15 (0.57, 2.34) |

CBRS: Conners Comprehensive Behaviour Rating Scales (parent report) – past 4 weeks. Emotional problem symptoms: MDD + GAD

† p<0.1 *p<0.05 **p<0.01 ***p<0.001
